# Correction: Dipyrrolyl-bis-sulfonamide chromophore based probe for anion recognition

**DOI:** 10.1039/d2ra90024f

**Published:** 2022-03-17

**Authors:** Namdev V. Ghule, Sheshanath V. Bhosale, Sidhanath V. Bhosale

**Affiliations:** Polymers and Functional Material Division, CSIR-Indian Institute of Chemical Technology Hyderabad 50060 AP India bhosale@iict.re.in; School of Applied Sciences, RMIT University GPO Box 2476V Melbourne 3001 Victoria Australia sheshanath.bhosale@rmit.edu.au +61399252680; Department of Organic Chemistry, School of Chemical Sciences, North Maharashtra University Jalgaon-425001 M.S. India

## Abstract

Correction for ‘Dipyrrolyl-bis-sulfonamide chromophore based probe for anion recognition’ by Namdev V. Ghule *et al., RSC Adv.*, 2014, **4**, 27112–27115, DOI: 10.1039/C4RA04000G.

The authors regret that an incorrect version of Fig. 1 was included in the original article. The correct version of Fig. 1 is presented below.

**Fig. 1:**
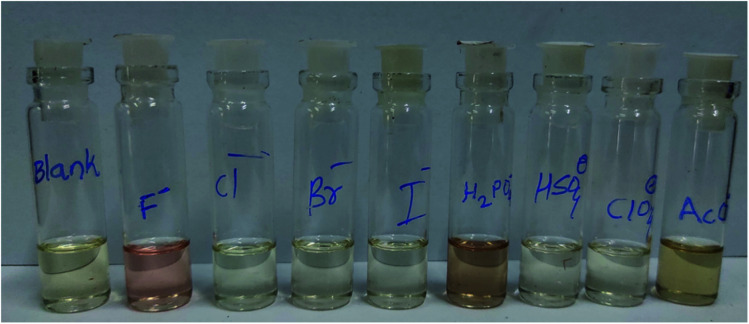
Color changes of receptor DPBS in chloroform upon addition of 5 equiv. of F^−^, Cl^−^, Br^−^, I^−^, H_2_PO_4_^−^, HSO_4_^−^, ClO_4_^−^ and AcO^−^ (tetrabutylammonium salts).

The Royal Society of Chemistry apologises for these errors and any consequent inconvenience to authors and readers.

## Supplementary Material

